# Synthesis in land change science: methodological patterns, challenges, and guidelines

**DOI:** 10.1007/s10113-014-0626-8

**Published:** 2014-06-06

**Authors:** Nicholas R. Magliocca, Thomas K. Rudel, Peter H. Verburg, William J. McConnell, Ole Mertz, Katharina Gerstner, Andreas Heinimann, Erle C. Ellis

**Affiliations:** 1 Department of Geography and Environmental Systems, University of Maryland, Baltimore County, 211 Sondheim, 1000 Hilltop Circle, Baltimore, MD USA; 2 Department of Sociology, Rutgers University, 26 Nichol Avenue, New Brunswick, NJ USA; 3 Institute for Environmental Studies, VU University Amsterdam, De Boelelaan 1087, 1081 HV Amsterdam, The Netherlands; 4 Center for Systems Integration and Sustainability, Michigan State University, Manly Miles Building, Suite 115, 1405 South Harrison Road, East Lansing, MI USA; 5 Department of Geosciences and Natural Resource Management, University of Copenhagen, Oster Voldgade 10, 1350, Copenhagen K, Denmark; 6 Department of Computational Landscape Ecology, Helmholtz Centre for Environmental Research – UFZ, Permoserstraße 15, 04318 Leipzig, Germany; 7 Swiss National Centre of Research (NCCR) North–South, Centre for Development and Environment (CDE), University of Bern, Hallerstrasse 10, 3012 Bern, Switzerland; 8 The National Socio-Environmental Synthesis Center, University of Maryland, College Park, 1 Park Place, Annapolis, MD USA

**Keywords:** Land-use change, Meta-study, Meta-analysis, Case studies

## Abstract

Global and regional economic and environmental changes are increasingly influencing local land-use, livelihoods, and ecosystems. At the same time, cumulative local land changes are driving global and regional changes in biodiversity and the environment. To understand the causes and consequences of these changes, land change science (LCS) draws on a wide array synthetic and meta-study techniques to generate global and regional knowledge from local case studies of land change. Here, we review the characteristics and applications of synthesis methods in LCS and assess the current state of synthetic research based on a meta-analysis of synthesis studies from 1995 to 2012. Publication of synthesis research is accelerating, with a clear trend toward increasingly sophisticated and quantitative methods, including meta-analysis. Detailed trends in synthesis objectives, methods, and land change phenomena and world regions most commonly studied are presented. Significant challenges to successful synthesis research in LCS are also identified, including issues of interpretability and comparability across case-studies and the limits of and biases in the geographic coverage of case studies. Nevertheless, synthesis methods based on local case studies will remain essential for generating systematic global and regional understanding of local land change for the foreseeable future, and multiple opportunities exist to accelerate and enhance the reliability of synthetic LCS research in the future. Demand for global and regional knowledge generation will continue to grow to support adaptation and mitigation policies consistent with both the local realities and regional and global environmental and economic contexts of land change.

**Electronic supplementary material:**

The online version of this article (doi:10.1007/s10113-014-0626-8) contains supplementary material, which is available to authorized users.

## Introduction

The need to adapt to and mitigate global environmental change has increased the demand to harness knowledge production for the needs of policy- and decision-making across global, regional, and local scales (DeFries et al. 2012; Turner et al. 2013). Policy-makers must consider the local realities of changing livelihoods and land-use patterns and their interactions with the regional/national and global contexts in which local change processes are embedded (An et al. 2005; Liverman and Cuesta 2008; Valbuena et al. 2010; Verburg et al. 2009). The production of generalized knowledge of the causes and consequences of local land change and their coupling with global and regional systems remains one of the fundamental challenges of land change science (LCS) (Parker et al. 2008; Rindfuss et al. 2008; Turner et al. 2007). This paper assesses current methods, motivations, and applications of synthesis research in LCS in an effort to advance the production of systematic knowledge of the causes and consequences of local land change globally.

Land change, consisting of both land-use and land-cover changes, is broadly conceived of as changes in terrestrial ecosystems resulting from human and environmental interactions, and their feedbacks overtime within land systems (Turner et al. 2007). Synthesizing generalized knowledge of regional or global patterns in local land changes and their drivers and consequences is challenging due to the complex, multiscale nature of land change processes. Local land-use options from which people choose are structured by general, broad-scale economic, political, cultural, and environmental processes (Geist and Lambin 2001; Lambin et al. 2001; Liverman and Cuesta 2008; Valbuena et al. 2010; Verburg et al. 2009). However, complex interactions between broad-scale processes and local land-owner and institutional decisions and actions can lead to widely varying outcomes that are often found to be highly context dependent (Parker et al. 2008; Rindfuss et al. 2004, 2007). Such cross-scale connections, more recently summarized under the term teleconnection (e.g., Liu et al. 2013), present substantial challenges for developing a general understanding of local land changes globally.

Generalization is further complicated by the varied research questions, scales of analysis, and theoretical frameworks used in LCS. Individual studies range from regional to global assessments spanning hundreds of years, to highly detailed local case studies at the level of individual communities in which the rich interplay of multiple biophysical and social factors is related to particular outcomes. For example, local land change case studies might cover a small geographic area (i.e., <100 km^2^) and focus on linking patterns of local land change to their ecological consequences, such as deforestation, or to causal factors such as micro-level social processes operating locally and/or regionally, such as land tenure relationships (Rudel 2008; Verburg et al. 2004). In contrast, regional to global assessments often use aggregated biophysical and socioeconomic data in conjunction with aggregated country-level statistics to link social factors to broad-scale patterns of land change (Rudel 2008; Verburg et al. 2004). These approaches span a variety of spatial and temporal scales, and may also be arrayed along a quantitative to qualitative continuum related to the scale and mode of inquiry used.

The disciplinary diversity inherent in land change research can produce varied interpretations and analyses of the same land change phenomenon. Many land change studies aim to quantify the ecological effects of specific land change processes, while others are concerned with the underlying factors causing land change. Among studies seeking causal explanations of land change processes, some explain emergent land change patterns based on geographic data (e.g., remotely sensed changes in forest cover), while others aim at understanding the socio-environmental cognitions underlying decision-making, such as the institutional contexts of land-use decisions. This difference is exemplified by the units of analysis which might focus on spatial units, such as pixels in remotely sensed imagery or political units, or on the individual decision-makers themselves (Overmars and Verburg 2005). In the first case, decision-making is inferred from observed spatial patterns, while the second approach focuses on decision-making processes or decision-makers as the object of study. Depending on the theoretical and disciplinary lens through which the analysis is conducted, explanations of the same land change patterns may be sought at the level of macro-scale ‘external’ drivers, at the micro-level of individual land change actors, or on some intermediate level in between (Hersperger et al. 2010).

To achieve general understanding of common and divergent land change processes and consequences across different places and times, synthesis of local land change knowledge collected at varying scales and with diverse methods is required. However, it is unclear which synthesis method or mix of methods is most appropriate for particular types of case-study data and for which synthesis purposes. Hence, this paper assesses the state of the art of synthesis methods employed in LCS to date and offers prospects for advancing synthesis research in LCS. An overview of current synthesis techniques distinguishes specialized meta-study methods from the broader family of approaches to synthesis. A meta-analysis is then conducted to assess the types of synthesis methods commonly used in LCS, taking into account the motivations for conducting synthesis research, the land change phenomena and world regions studied, and the disciplines from which the synthesis studies originate. Finally, the challenges and potential biases inherent in LCS synthesis approaches are described, and suggestions are made for advancing LCS synthesis toward the ultimate goal of producing generalized knowledge of the local realities of land change within the context of global environmental change.

## Synthesis methods in LCS

Before describing specific synthesis methods used in LCS, it is useful to differentiate and define generalization as an objective, synthesis as a research approach involving a broad family of methods, and meta-studies as special cases of synthesis methods. A central challenge of land change research is to connect observations of local change to more general causes and consequences and move beyond case-specific explanations and the ‘variance of place’ (Turner et al. 2007). *Generalization* of the causes and/or consequences of land change at regional to global scales from local observations is thus a main objective. *Synthesis* is a research approach that draws upon and distills many sources of data, ideas, explanations, and methods in order to accelerate knowledge production beyond that of less integrative approaches (see ‘synthesis’ at http://sesync.org/glossary/). *Meta*-*studies* are specific synthetic methods that distill the findings of many narrowly focused analyses (i.e., ‘cases’) to produce knowledge that is more generally applicable than may be derived from a single case. Synthesis methods in LCS are used to build knowledge of general patterns across many cases to connect local observations of land change to more widely applicable explanations of the causes and/or consequences at regional and global scales.

**Fig. 1:**
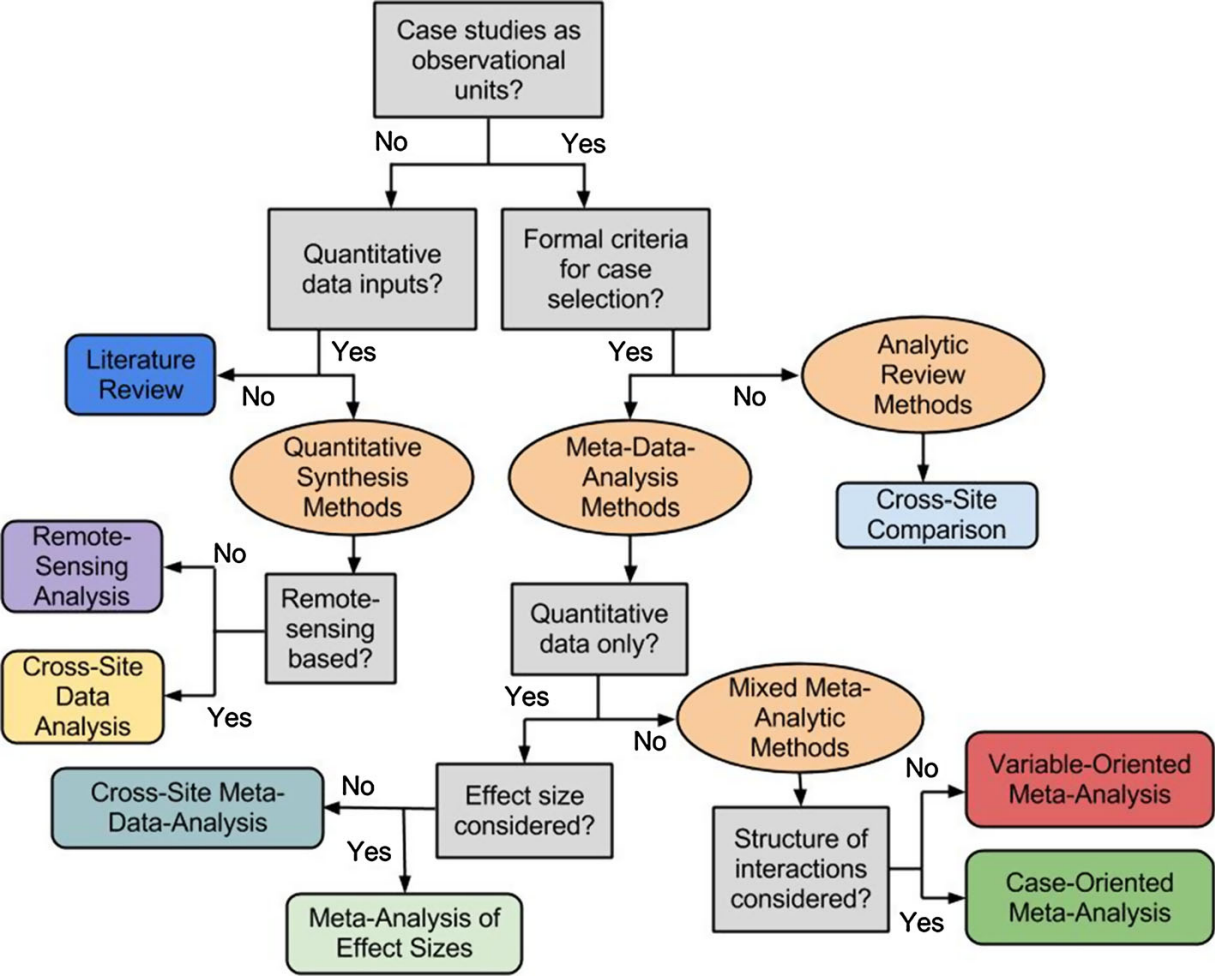
Heuristic tree to classify commonly used synthesis methods found in LCS

To navigate the breadth of disciplines, data types, and research questions applicable to LCS, a typology of synthesis methods using a heuristic tree is presented in Fig. 1. This typology will organize the following description of synthesis methods in LCS, as well as provide the classification scheme for the analysis reported in sections ‘A meta-analysis of synthesis methods in LCS’ and ‘Results.’ Table 1 provides corresponding descriptions, objectives, and examples of each synthesis approach commonly used in LCS. The broad synthesis domain classifications used here are based on the typology and definitions of systematic analyses in the PRISMA statement (Liberati et al. 2009)—which provides reporting guidelines for case-based systematic reviews and meta-analyses of health care interventions and outcomes—while specific synthesis approaches are classified according to methods common to LCS.

**Table 1:** Descriptions, objectives, and examples of synthesis (S) and meta-study (M) methods used in LCS

Synthesis domain	Synthesis method	Definition	Objective	Example
Synthesis				
Literature review	Literature review	A synthesis of concepts, data, and/or arguments from an unsystematically selected collection of theoretical and empirical sources	Summarize the state of knowledge relevant to a particular research question based on published literature	Meyfroidt and Lambin (2011)
Quantitative synthesis methods	Remote-sensing analysis	A synthesis of land change quantities obtained from remote-sensing data	Synthesize patterns of land change based on spatial data, and quantify central tendencies of those patterns	Brown et al. (2005)
	Cross-site data analysis	A statistical analysis identifying patterns across aggregate variable data (i.e., number-crunching)	Characterize the central tendencies of variables across sites	Winters et al. (2009)
Meta-study				
Analytic review methods	Cross-site comparison	A synthesis of an unsystematically selected collection of cases studies	Comparison of case studies spanning multiple sites to identify common outcomes, explanations, and/or system structures	Cramb et al. (2009)
Meta-data-analysis methods	Cross-site meta-data analysis	A statistical analysis (e.g., regression) across data values reported in systematically selected case studies	Derive quantitative relationships/model of factors correlated with land change outcomes; ex-post, data-driven variable coding system	Angelsen and Kaimowitz (1999)
	Meta-analysis of effect size	A statistical analysis (e.g., regression) of the magnitude of effects of land change conducted across case studies	Quantify the effects of land change under different conditions	Rey Benayas et al. (2009)
Mixed meta-analytic methods	Variable-oriented meta-analysis	A statistical analysis identifying cause-effect links between coded variables that run across cases	Derive quantitative relationships/model of factors correlated with land change outcomes; ex-ante, theory-driven classification system	Geist and Lambin (2001)
	Case-oriented meta-analysis	An analysis of coded data in which the relationships between coded variables are analyzed within and across cases	Derive structural relationships/model of factors leading to land change outcomes; ex-ante, theory-driven classification system	Rudel et al. (2005)

The core definition of meta-study presumes an analysis conducted across prior analyses or ‘studies’ that constitute ‘cases’ of a common phenomenon (Rudel 2008). The first classifying characteristic in this typology, then, is the use of cases as the observational unit, which distinguishes meta-study approaches from both literature reviews and fully quantitative synthesis methods. Next, the presence of formal case selection criteria separates analytic reviews and meta-studies. Case selection criteria must be described in sufficient detail to allow the reselection of the final collection of cases. Specifically, formal case selection criteria must include at a minimum (1) where cases were obtained (i.e., search engine (e.g., Web of Science)) or at least types of literature (e.g., peer-reviewed journal articles, gray literature, etc.), (2) which variables and/or relationships between variables were extracted from cases, and (3) why cases were excluded (e.g., due to data quality/type, time frame, or analysis performed). If these elements do not characterize the selection of the cases for the review, then we refer to the analysis as an analytic review method. Analytic review methods lack comprehensive case coverage, due to either limited data availability or no intention of representing a particular population of potential cases.

General synthesis approaches, including literature reviews and quantitative synthesis methods, can be applied when observations of the land change topic of interest are present in specific bodies of literature and/or aggregate statistics. Literature reviews synthesize concepts, opinions, and/or arguments across a set of publications to test a particular theoretical assertion, where the selected publications are not a particular manifestation of a common land change process (i.e., cases). Quantitative syntheses are analyses conducted across a set of data points, rather than cases, in an attempt to capture the central tendency and variations in the data. Although such broad synthesis methods are neither systematic nor case-based, they have contributed significantly to our understanding of patterns in land change across sites and are therefore included in the family of synthesis methods used in LCS.

 By contrast, *meta*-*studies* represent systematic review and synthesis across detailed cases of a generic phenomenon. A case represents a set of observations of the phenomenon of interest meeting predefined criteria (e.g., Seto et al. 2011) pertaining to the method of observation (e.g., field based) and research question. Meta-studies aim to obtain a sample of cases that captures the variability of land change outcomes observed for the phenomenon or land system of interest by systematically searching for case studies from available literature. Thus, meta-studies may be thought of as formalized reviews of sets of case studies. *Meta*-*analyses* are forms of highly structured meta-studies that utilize more standardized and explicit methodologies to statistically compare parameter values and their variance within and across systematically selected case studies. To compare statistical variance across case studies, the data requirements are quite stringent: All case studies must report data on the same variables, using similar instruments and comparable settings, and provide quantitative measures of variance in effects that can be assessed relative to variance across studies (Gurevitch and Hedges 1999; Rudel 2008).

The strengths and limitations of each synthesis technique are provided in Table 2. Given the breadth of research questions and data used in LCS, no single synthesis method will be appropriate for all situations. Both the intent of the researcher and the type and quality of the data available for analysis will contribute to the choice of synthesis method used and robustness of their results. However, meta-analysis offers the most statistically robust strategy for synthesizing observations across case studies, exemplifying rigorous, quantitative techniques for developing general knowledge from case studies.

**Table 2:** Strengths and limitations of synthesis methods used in LCS

Synthesis method	Strengths	Limitations
Synthesis		
Literature review	Highlight targeted set of findings to frame research questions	Article selection may not be systematic Uncertain unit of analysis, not well suited for (quantitative or qualitative) analysis
Remote-sensing analysis	Produces quantity and spatial extent of land change	Limited by spatial data availability Site selection may not be systematic Description of observed patterns only, no causal explanation possible
Cross-site data analysis	Quantification of broad patterns across variables related to the causes and/or consequences of land change	Often restricted to aggregated data Article selection may not be systematic Not well suited to explore outlier cases Description of observed patterns only, no causal explanation possible
Meta-studies		
Cross-site comparison	Enables comparative analysis, identification of common processes, outcomes	Article selection based on the ability of the case to illustrate a particular phenomenon, may not be systematic
Cross-site meta-data-analysis	Case selection criteria explicit, systematic Quantified relationships between potential causes/consequences and land changes Quantitative and qualitative data	Structure of factor interactions uncertain, restricted to correlative relationships
Meta-analysis of effect sizes	Case selection criteria explicit, systematic Uses a common measure (i.e., effect size) to compare cases Identification of quantitative patterns in the effects of land change across sites	Restricted to quantitative data Statistical power is limited by sample size and incomplete reporting of analyzed cases
Variable-oriented meta-analysis	Case selection criteria explicit, systematic Quantified relationships between land changes and other variables Quantitative and qualitative data Coding grounded in theory	Structure of factor interactions uncertain, restricted to correlative relationships Capture central tendency of land change causes/consequences, but may obscure outliers
Case-oriented meta-analysis	Case selection criteria explicit, systematic Structure of interactions considered explicitly Quantitative and qualitative data Coding grounded in theory	Often limited by the absence of ‘no change’ cases Case coding often requires simplified descriptions of driver–impact relationships, may lose process-level detail

### Variable- and case-oriented approaches to meta-analysis

The multicausal nature of land change often requires meta-analytic techniques that can combine both quantitative and qualitative data, which is done in LCS through either variable- or case-oriented approaches. The most common approach to coding is the variable-oriented approach that uses statistical routines to identify cause-effect links between variables that run across the assembled studies. Variable-oriented statistical routines like regression match up well with the goal of generalization because they excel at elucidating the central tendencies in a data set. Studies of patterns of joint causation leading to land change often conduct frequency analyses of variable occurrences (e.g., Geist and Lambin 2004; Keys and McConnell 2005). Other studies are able to analyze the probability of occurrence of a land change phenomenon using regression models (e.g., Turner et al. 1977). Variable-oriented analyses can produce fine-grained estimates of the average size of one variable’s effect on another variable across a set of studies. Case-oriented approaches, such as that conducted by Rudel (2007) examining the multiple factors contributing to deforestation, provide a useful complement to the variable-oriented analyses in that they identify sets of similar cases associated with the outcome of interest. Rather than focusing on the cause–effect links between individual variables, case-oriented approaches emphasize multiple conjunctural causation in which a combination of conditions cause the outcome of interest (Ragin 1987). Case-oriented analysts sometimes use a sophisticated implementation of set theory employing Boolean algebra referred to as qualitative comparative analysis (QCA) to identify patterns of associated conditions that run across the cases studies in the meta-analysis.

Researchers tend to use variable-oriented approaches when the number of case studies is large, because the possible number of comparisons between cases becomes unwieldy for case-oriented approaches (Rudel 2008). However, this rule of thumb does not always apply, and the choice of method frequently depends on the researcher’s goals. If he/she is particularly concerned with ‘deviant cases,’ ones that depart from the central tendency in the literature, then QCA might be the preferable analytic tool because it does not ignore outlying cases in the way that statistical analyses would. Rather, the unique conditions that have shaped an outcome are listed, which invites the researcher to develop an explanation for the unusual outcome in this case. Because they present alternative outcomes, these deviant cases can point the way to policy initiatives that could alter patterns of change in human–environment relations. These differences in emphasis between variable- and case-oriented approaches underscore Borenstein’s observation (2009) that different situations call for different meta-analytic methods.

### Meta-analyses of effect sizes

Due to the great variety of research questions in LCS, the statistical methods employed also differ widely. Meta-analyses of effect sizes, a special class of meta-analyses typically used to investigate the consequences of land change on the environment, are used to investigate treatments or gradients of land-use and their effects on a specific response variable (e.g., De Schrijver et al. 2011; Guo and Gifford 2002; Sodhi et al. 2009). The products of such meta-analyses are quantified effect sizes and their variance, which can be estimated from the data given in the case studies. General trends of effects can be analyzed, as well as the determinants of variance, which usually require special statistical methods designed for meta-analysis of effect sizes and their variance (Gurevitch and Hedges 1999; Nakagawa and Santos 2012). Most often used in ecology (Arnqvist and Wooster 1995), meta-analyses of effect sizes are the most statistically rigorous meta-analytic methods used in land change research.

## A meta-analysis of synthesis methods in LCS

### Acquiring and selecting LCS synthesis studies

The first task is to clearly define the phenomenon under study and a set of keywords that bound the population of cases under consideration. In this study, we assess the scope of and trends in synthesis efforts contributing to LCS over time. A target set of 19 known meta-studies was first identified to represent the full expected range of synthesis methods and research topics present in LCS. Keywords are selected from these meta-studies such that all target meta-studies appear in the search results. The list of target meta-studies and the exact keywords and search terms are reported in Appendix 1 of Supplementary Material. Candidate synthesis efforts in peer-reviewed journal articles, book chapters, and reports were then identified using the Web of Science^®^.

A synthesis study is selected for analysis if it is deemed relevant to LCS and of sufficient geographic extent to provide generalized knowledge. A synthesis study is considered relevant to LCS and included in the meta-analysis if the objectives of the synthesis are to understand:

1. Human modifications and appropriations of natural processes as *causes* of observed land-cover change;
2. How the *consequences* of land-use directly affect natural and/or human system outcomes;
3. A combination of (1) and (2).

This is by no means a comprehensive definition of LCS, but it does constrain the meta-analysis to synthesis studies that address the direct role people have in driving land change—which is a fundamental tenet of LCS (Rindfuss et al. 2008; Turner et al. 2007). Similarly, synthesis studies were excluded if humans are not one of the direct causes of observed land change (i.e., climate change is considered an indirect human cause of land-cover change).

In addition, the range of synthesis studies included is further narrowed by stipulating that the spatial scale of the study must be sufficient for interregional comparison and/or global analysis. As operational criteria, a given study’s findings must:

1. Be generalizable beyond the set of sample cases analyzed or can be used to describe regional trends globally;
2. Be representative of a globally or regionally relevant process; and
3. Not be constrained by political boundaries alone.

The keyword search returned 3,672 publications. Of these, 181 published articles met the above criteria. A complete list of the studies and their coding is provided in Appendix 2 of Supplementary Material.

### Case coding

The next step was to code each synthesis study. Cases were first coded by synthesis method according to the heuristic tree in Fig. 1. Next, each case was coded for the researcher’s apparent purpose for conducting the synthesis study using four categories: ‘theory,’ ‘praxis,’ ‘model,’ and ‘policy.’ These broad categories of stated objectives emerged consistently during several rounds of re-coding. Although *theory* is a distinct category, all synthesis efforts are used for theory-building to some degree. Thus, in this context, the category *theory* is used to describe synthesis studies that are conducted solely for the purpose of advancing or testing existing theory, and/or building new theory, usually by testing a particular hypothesis or evaluating the state of knowledge. The other categories describe studies which also contribute to theory, but have other primary purposes. Studies categorized as *praxis* explicitly use synthesis to identify further research needs and/or address inconsistent case-study methods. *Model* studies use synthesis methods to design, test, and/or parameterize analytical or simulation models, which can then be applied at a level of abstraction or spatial or temporal extent beyond the local case-study. Finally, *policy* studies primarily aim to synthesize knowledge of local outcomes to evaluate the effects of existing policy across locations and/or aggregate data for creating new policy.

Synthesis studies are additionally coded by land change topic area, world region, discipline, and explanatory focus. The land change *topic area* describes the processes being investigated, and meta-studies often address interrelated topic areas (e.g., deforestation and agricultural expansion). The *geographic extent* of each meta-study is roughly coded by world region based on the convention of Geist and Lambin (2001) for coding case studies of land change, along with the category of ‘global.’ Although a more nuanced geographic categorization may be desirable, many of the synthesis studies analyzed did not provide sufficient geographic descriptions, and we discuss our results in light of this limitation. *Discipline* is coded based on the disciplinary affiliations of the journal in which the meta-study is published. A standard set of disciplines was taken from www.journalseek.net, and when multiple disciplines were found for a given journal, the meta-study is coded as ‘interdisciplinary.’ Finally, each meta-study was coded by the *explanatory focus*, or objective, of its organizing research question. LCS meta-studies tend to investigate how human modifications and appropriations of natural processes are causes of observed land change, or how the consequences of human use of land directly affect land system outcomes. Thus, a meta-study can be coded as either strongly causal or strongly consequential, or a combination thereof.

Coding was done by a multiperson team in two phases. The first phase involved choosing and iteratively coding practice sets of ten randomly selected studies from the full set. This phase served to increase intercoder reliability by comparing interpretations and coding agreement of studies across team members, coordinate inclusion/exclusion criteria, and formulate and negotiate operational definitions for each coding category. Once sufficient coding agreement was achieved, the next phase divides the full set of meta-studies between team members. Periodically, coding definitions were adjusted in light of challenging cases, which required recoding some or all cases. Overall, intercoder reliability was high with above 90 and 80 % agreement between coders for the processes of including/excluding and coding articles, respectively.

## Results

**Fig. 2:**
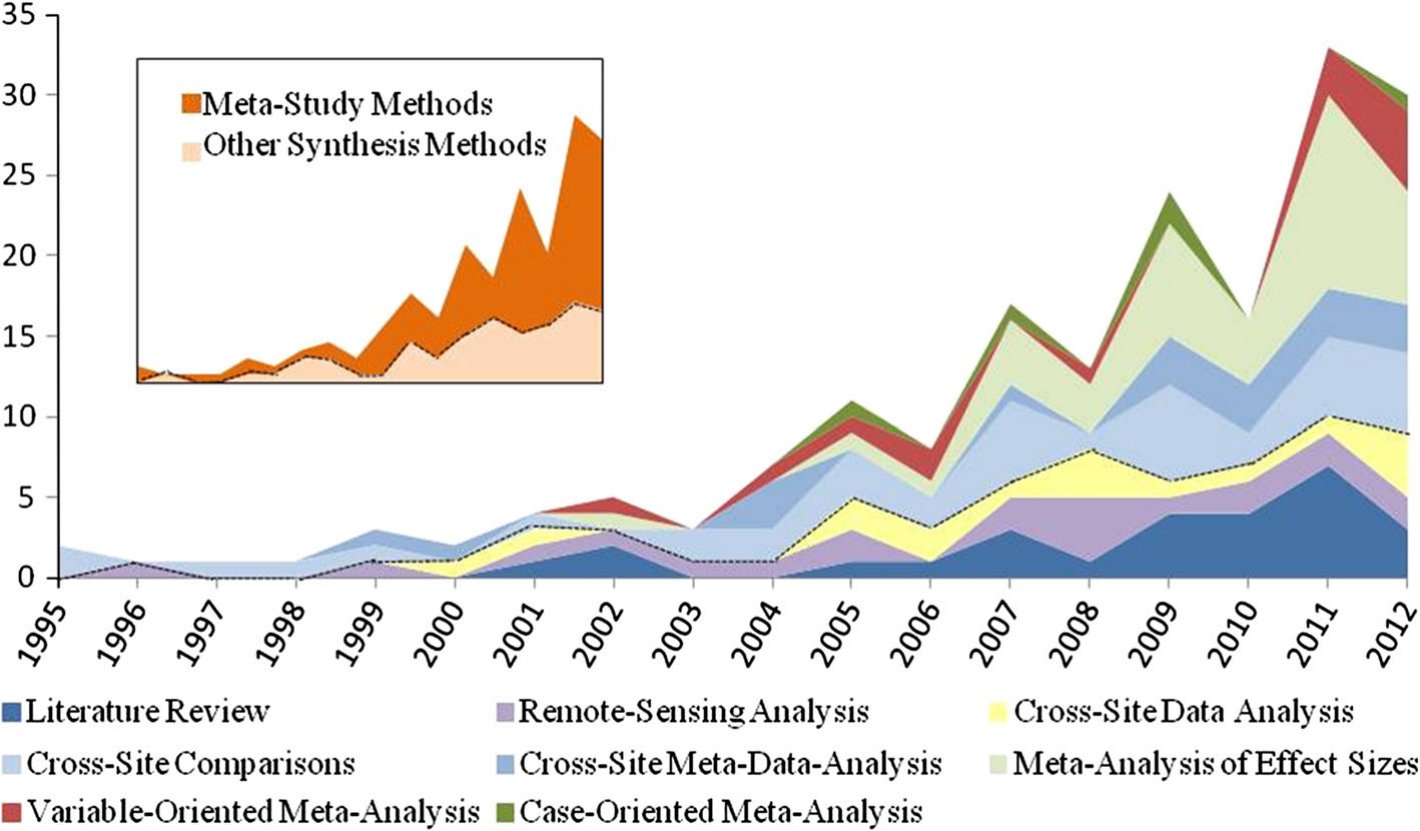
An overall increase in general synthesis and meta-study research was apparent from 1995 to 2012, with meta-analyses of effect sizes and literature reviews of land change becoming relatively more abundant

The 181 articles selected for analysis were published between 1995 and 2012, during which time an overall increase in synthesis efforts was apparent (Fig. 2), particularly in the number of meta-studies conducted (see inset of Fig. 2). Meta-analysis of effect size was the dominant meta-analytic methodology, while the conventional literature review was the most commonly used general synthesis method.

**Fig. 3:**
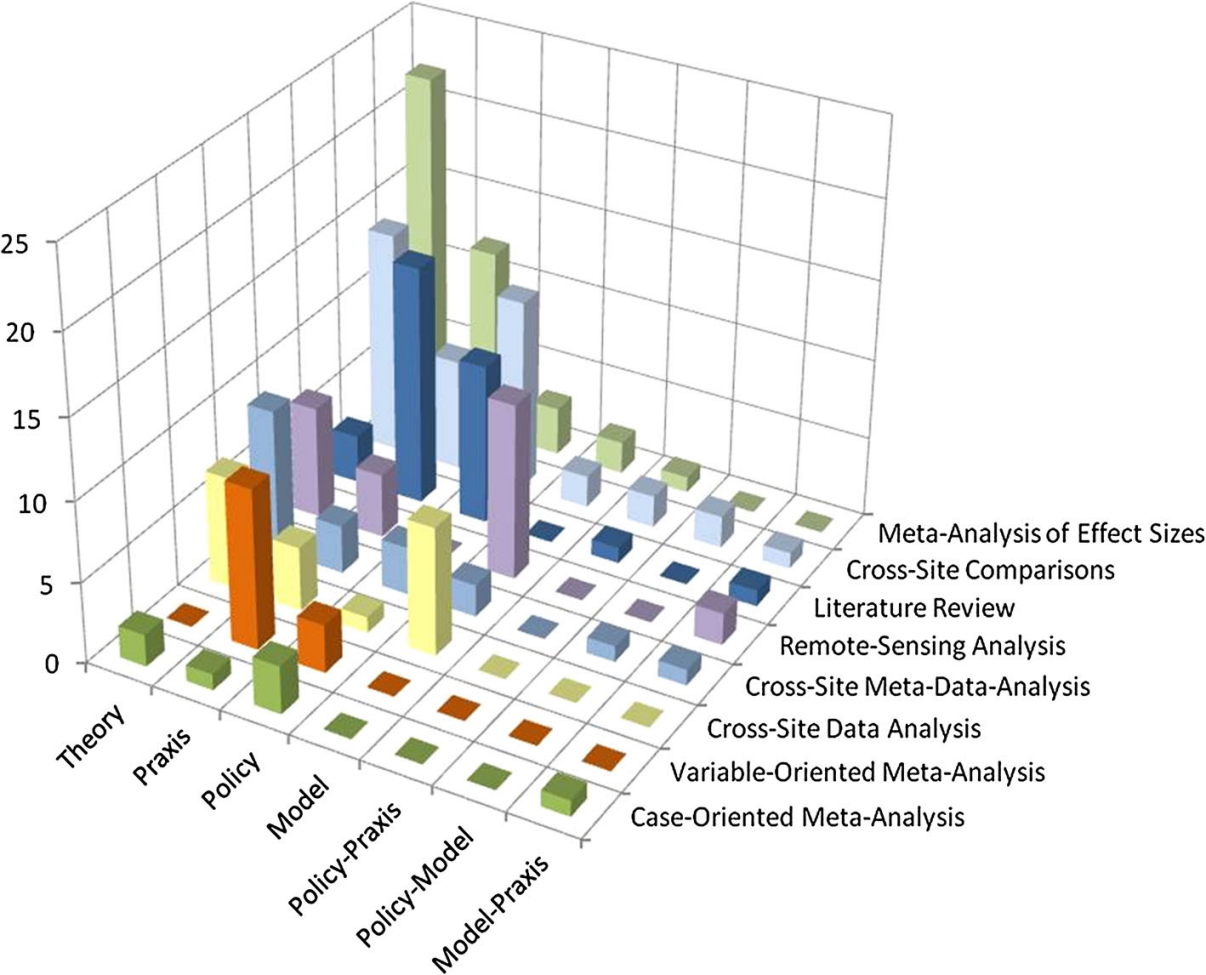
Relationships between the method used and purpose of each synthesis study. Note that theory is implied in every category, and the order of synthesis methods has been changed to avoid occultation

Some relationships between the purpose of the synthesis study and the methodology used were also apparent (Fig. 3). Synthesis studies overall were predominantly used either for advancing, testing, and/or building new theory (*n* = 64) or in combination with efforts to improve praxis (*n* = 52). Policy evaluation (*n* = 38) and model building (*n* = 27) were less frequently invoked as the motivation for synthesis research. However, 11 studies were motivated by a combination of two or more of these themes.

Evaluation of policy outcomes and research praxis was often pursued via less formal meta-study and synthesis methods, such as literature review and cross-site comparisons. Although, praxis was also a frequent concern for meta-analyses attempting to compare quantitative measurements across cases, such as meta-analyses of effect sizes and variable-oriented meta-analyses, because inconsistency across sample sizes and data collection methods often limited the statistical power of such methods. In contrast, quantitative synthesis methods, such as cross-site data and remote-sensing analyses, were the dominant means for assisting model building, reflecting the quantitative demands of model parameterization. Finally, meta-analyses of effect sizes and cross-site comparisons were the primary choices of researchers solely seeking to advance theory. In these instances, a particular cause–effect relationship was hypothesized, and the synthesis study was used to describe variability in the hypothesized relationship in a more or less systematic way (i.e., meta-analysis of effect sizes and cross-site comparisons, respectively).

**Fig. 4:**
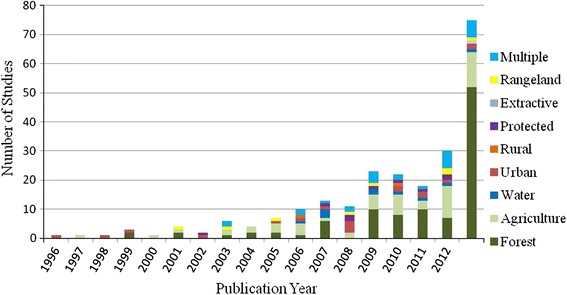
Trends in categories of land change phenomenon in synthesis studies published between 1995 and 2012

Land change phenomena studied in these articles (Fig. 4) were dominated by forest-related (i.e., deforestation/afforestation) and agriculture-related (i.e., agricultural intensification, expansion, and/or abandonment) topics. These topics remained foci of land change research as their representation increased proportionally with all types of synthesis research overtime. Land change in urban (i.e., urban growth or shrinkage), rangeland (i.e., pasture degradation or desertification), and protected area (i.e., conservation areas) contexts made up relatively small, but consistent shares of synthesis research over the past 18 years. Synthesis of water-related research (i.e., use of, or impacts on, water resources from land-use) was present in seven of the last 8 years and appeared to be an emerging area of emphasis. An increased interest in investigating the impacts of multiple, interacting land-uses was also evident, as studies considering multiple land change phenomena become more abundant overtime.

**Fig. 5:**
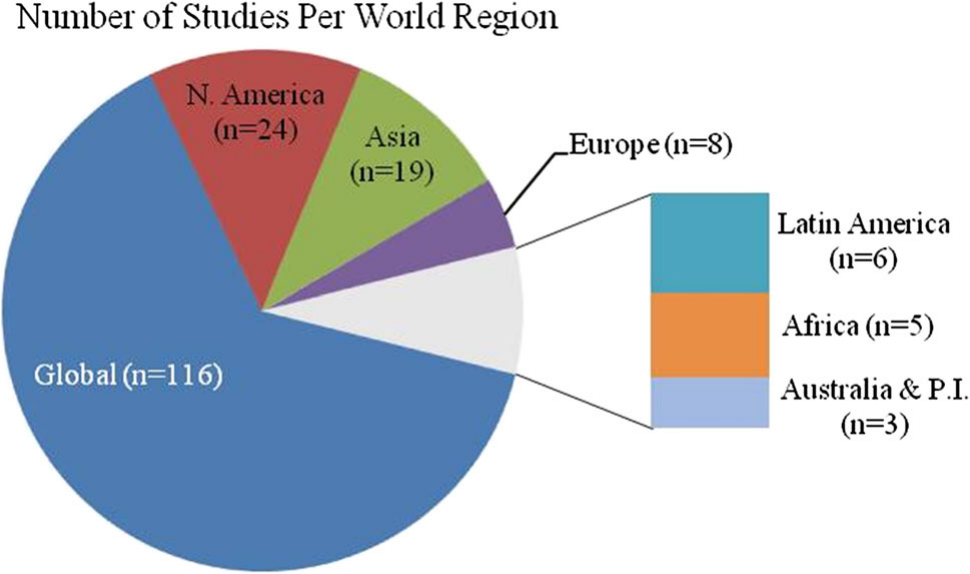
Geographic extent of synthesis and meta-study articles

According to our selection and coding criteria nearly, two thirds of the studies (64 %) examined a geographic extent that could be considered ‘global’ (Fig. 5). The remaining studies were contained within particular world regions, although most studies did not provide sufficient geographic descriptions to more precisely characterize the region of study. Among the regional studies, North America received the most attention, followed by Asia and Europe.

**Fig. 6:**
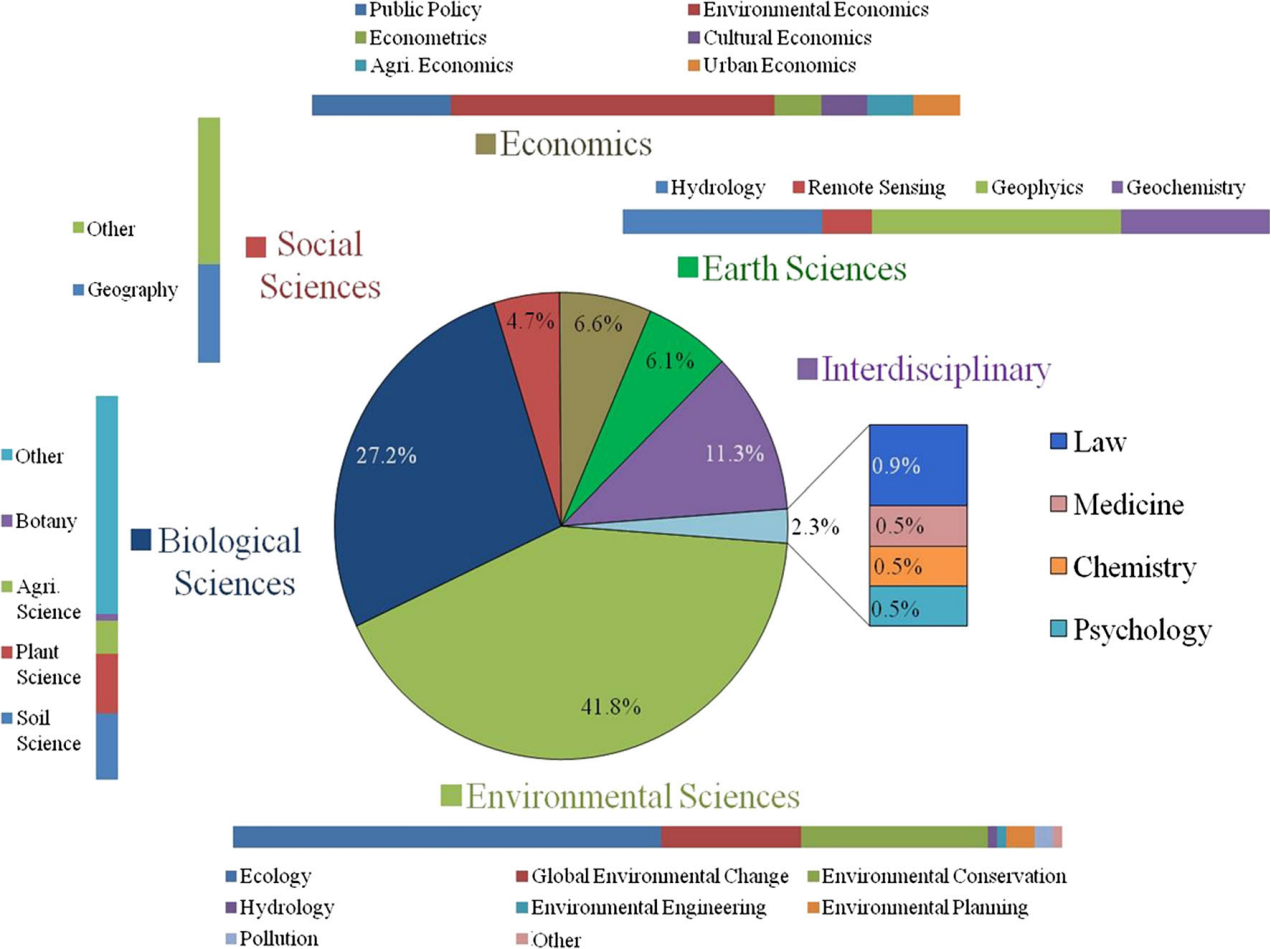
Diversity and representation of major disciplines and subdisciplines engaged in synthesis research of the causes and/or consequences of land change

Roughly 80 % of synthesis and meta-study research investigated the consequences of land-use change, while the remaining studies examined the causes or a combination of causes and consequences of land change phenomenon. This inequality was likely related to the disciplines contributing to land change research and their respective methodological and topical foci, with more than three quarters of studies coming from the biological, earth, and environmental sciences. However, the multidisciplinary nature of land change research was clear from the findings. Figure 6 illustrates the representation of each discipline and subdisciplines among the studies analyzed. Nearly 42 % of studies were published in journals classified as environmental science, with the fields of ecology, global environmental change, and environmental conservation particularly well represented. The next most common group was the biological sciences. Although the most abundant subcategory in biological sciences was described as miscellaneous, these journals most often published research on the effects of land-use on biodiversity. The third highest proportion was from studies published in journals classified in more than three major disciplines and thus categorized as explicitly interdisciplinary. Finally, the broad disciplinary range of LCS was apparent with nearly 5 % or greater contributions from each social sciences, economics, and earth sciences.

## Discussion

### Importance of synthesis research in LCS

Global environmental change influences and is influenced by local land change, and synthesis research methods are an important tool used by many disciplines for building systematic knowledge of land change phenomena. A primary motivation for conducting global synthesis, which was common across both land change phenomena and disciplines, was the need to integrate fragmented local observations to create broader-scale, general knowledge of variations in patterns of land change in order to advance theory, with most studies focused on investigating the consequences of land change. This is likely a reflection of the fact that the consequences of land change, such as changes in biodiversity or soil carbon, are directly measurable and inherently better suited for meta-analysis. Field measurements can be collected with standardized data collection protocols, statistical procedures are generally consistent across case studies, and quantitative results are easily interpretable. Additionally, the mitigation of negative land change consequences is often the focus of land management and policy initiatives (e.g., REDD+) and is thus a primary motivation for synthesis efforts (van Vliet et al. 2012). In contrast, the causes of land change are often not directly observable nor easily measureable. This may require integration of both quantitative and qualitative data, which complicates the interpretation and standardization of case-study findings needed for synthesis.

Agricultural and forest change have traditionally been foci for synthesis research, but both subjects have seen renewed interest owing to the recent global economic and environmental implications of biofuel production and carbon offset/sequestration. In both instances, regional to global costs and benefits are incurred but with differential local socio-economic and ecological impacts. For example, Achten and Verchot (2011) conducted a cross-site comparison to assess whether potential greenhouse gas emission reductions from the use of biofuels could be overwhelmed by local emissions associated with land-use changes from biofuel production. Similarly, Ziegler and colleagues (2012) performed a meta-analysis to assess the impacts of land-use transitions away from swidden agriculture to cash crop and biofuel production within the context of REDD+ policies. More broadly, several studies were undertaken to assess the magnitude and direction of regional to global soil carbon changes in response to local deforestation/afforestation with the aim of quantifying carbon sequestration potential from forestry practices (e.g., Guo and Gifford 2002; Li et al. 2012).

An emerging research area gaining interdisciplinary attention was the link between water resources and the causes and/or consequences of land change. Water–land-use interactions were investigated in a number of ways including surface water contamination, groundwater depletion, agricultural production, resource capture by the elite, and ecosystem conservation (Srinivasan et al. 2012). For example, Srinivasan and colleagues (2012) performed a case-oriented meta-analysis across 22 case studies describing the state of water resource outcomes from which they identified distinct ‘syndromes’ and the factors creating them globally. Despite the multitude of interactions between land-use and water quality and quantity, little is known about the state of water resources globally. The upward trend in synthesis and meta-study research around water and land-use interactions observed in the last decade suggests that such methods are essential for integrating local evidence to describe global patterns of water resource use and change.

### Caveats

A number of caveats apply to the collection of studies we analyzed. First, this can be considered neither a complete nor a random selection of land change synthesis studies. To ensure replicability, we used a standard search engine that was restricted to English language publications in the peer-reviewed literature. However, this introduced potentially important selection bias by excluding non-English studies and ‘gray literature,’ as well as limiting the analysis to the most readily available synthesis studies. Second, disagreements between coders about the inclusion/exclusion and coding of studies were unavoidable. Iteratively coding a control set of studies built common understanding and interpretations and increased intercoder agreement. Finally, descriptions of the geographic extent of syntheses and meta-studies were generally poor. Thus, we had to accept an author’s statement that a systematic synthesis or meta-analysis described a global pattern in local land change, yet there was no way to assess whether a globally or regionally representative selection of case studies was used. The first two limitations are common to meta-analytic methods. However, the third is a particular challenge inherent to land change research, which often must navigate both geographic and interpretational complexities in the multidisciplinary case-study literature.

## Challenges and prospects for advancing synthesis in LCS

Several characteristics distinguish synthesis efforts, particularly meta-analyses, in LCS from those in other disciplines, such as medical research, ecology, and economics. First, inputs are often a mix of quantitative and qualitative data. This mix is often determined by the degree of compatibility of data and sampling methods across case studies, as well as the extent to which the interpretation of case-study authors’ findings is integrated into the meta-analysis. Second, some meta-analytic techniques used in LCS, such as QCA, use the structure of interactions between variables within a case as the unit of analysis (i.e., case-oriented meta-analysis), which yields a qualitatively different explanation of variation across cases than meta-analyses of quantitative data only. Finally, and perhaps most important, standards for conducting a land change case-study are even less agreed upon in LCS than in other contexts due to the diversity of contributing disciplines, which can lead to substantial inconsistencies in data types and observational instruments across case studies.

### Interpretability and standardization

Synthesis methods require standardization—or at least harmonization—of relevant data across cases. In the context of LCS, where the blending of quantitative and qualitative data is common, standardization often entails identifying a robust array of possible cause–effect relationships involving the land change phenomenon of interest and providing operational definitions for each variable that can be identified in the articles and implemented as codes. If the number of case studies in a meta-analysis is large, the researcher may have to train others to perform the coding based on a predefined set of variables. When disagreements occur in the coding of a variable in a particular case, a discussion between the coders ensues about ways to classify or measure the presence or absence of a particular variable in a case. Alternatively, these discussions may expose an ambiguity in the variable as it is formulated and/or reported. A kind of ‘progressive contextualization’ (Vayda 1983) occurs in which the analyst explores and then explicates the links between land changes and the larger contexts of the change. Through these iterative procedures, the analyst may become aware of new patterns in the data, often having to do with the contexts of the case studies.

Generally, the more vaguely defined or reported a variable, the more likely it is that coders interpret a particular case differently and data standardization across cases will be difficult to establish. Interpretability and standardization challenges arise from the inherent difficulties in comparing case studies that are conducted with different objectives, use diverse approaches, and methodologies, and rely on varying levels of information and empirical evidence for results. Unambiguous empirical evidence for cause–effect relationships in land change studies is difficult to establish as feedback mechanisms and multiple drivers easily confound relationships, necessitating highly contextualized explanation. Thresholds of solid evidence and forceful argumentation are not clear-cut, and although quantitative methods often appear more convincing in describing relationships, qualitative methods are often more powerful for analyzing causality (Rudel 2008). Substantial variability may exist across case-study findings, which can only be disentangled if the primary data are available.

The spatial scale and extent of analysis within case studies are additional sources of interpretation and standardization issues, and can bring into question the spatial validity of synthesis and meta-study findings. This stems from incomplete or ambiguous geographic descriptions of the study sites used in case studies. A high-quality geographic description of a case-study site depends not only on the precision of the geographic details provided (e.g., geo-referenced maps, geographic coordinates, and/or text descriptions), but also on the clarity of the relationship between the geographic site and the reported data, and the degree to which subsequent users of the case-study are able to accurately interpret the geography and global context of the study site in a geographic information system (e.g., to map study site coverage across world regions). Frequently, geographic descriptions of case-study sites are missing at least one of these elements. Geographic descriptions are commonly provided in the form of maps, but are often not represented with the precision needed to make direct connections between reported data and the boundaries of the study area. For example, local land change case studies are often conducted at the village level and represented as points on a map. While the inhabitants of a village (or county, municipality, or other administrative area) and their land-use decisions might be central to the research, the land change patterns represented in such studies might precisely conform to a village boundary, extend across village boundaries, or represent some undocumented subset of land-use patterns within these boundaries. In general, for case studies of geographic entities above the size of small field plots, geographic point locations are incapable of capturing the geographic context and variability of land change patterns or processes typical in most land change case studies. These issues of geographic representation make it difficult to assess which part or parts of Earth’s land are actually represented by the results of a given synthesis and cast doubt on how generally applicable findings may be to the global or regional patterns of local land change processes.

The aggregation of individual case-study data required for synthesis may therefore suffer from the inherent problem of attempting to compare the incomparable. As a first priority, strengthening the case-study data reporting standards in LCS to create forms useful for cross-study synthesis (metadata) is essential, especially if this can be supported by enhanced tools for data sharing, searching, and synthesis across studies (Wolkovich et al. 2012; Agarwal et al. 2010; Ellis 2012). The geographic location and extent of individual LCS studies are especially critical to understanding global context and relevance of case studies for synthesis research and should be described using standard geographic data, such as Google Earth.kml, which has already become an option at Elsevier journals (e.g., Jetz et al. 2012; Karl et al. 2013; Martin et al. 2012; Van Vliet et al. 2012). A complementary approach—revisiting older case-study sites to create longitudinal datasets—would provide more empirically solid data compared to ‘snapshot’ studies that rely on recall and/or predictions. A recent example, following on a global meta-study on swidden cultivation (van Vliet et al. 2012), is a compilation of 8 longitudinal case studies on the consequences of swidden change (van Vliet et al. 2013).

Adoption of other non-spatial standardized observational instruments and data reporting standards, like those prescribed by the diagnostic institutional analysis and development framework for analyzing socio-ecological systems developed by Ostrom (2007) or the FAO’s Land-Cover Classification System (Jansen and Di Gregorio 2002), would also facilitate cross-comparison and analysis of local land change case studies. However, the utility of such metadata descriptors tends to depend on the user and specific synthesis effort. While greater metadata detail would appear to offer greater opportunity to support synthesis, we acknowledge that more detailed meta-knowledge systems are time-consuming for data producers to apply to their studies, but firmly believe their use is crucial to the advancement of LCS. It is worth noting that Ostrom’s well-known work on the governance of forest commons—the International Forestry Resources and Institutions (IFRI) project (Wollenberg et al. 2007)—was supported by FAO, following on the success of her prior cross-site work on irrigation systems (Tang^1992^). Programs underwriting comparative work, such as the US NSF’s Research Coordination Network program, may build on the IFRI model to support the sorts of standardized studies necessary for rigorous testing and development of land change theory.

The desire for sufficiently compatible data to support comparative land change analysis is not new, extending back at least two decades, including an edited volume on the comparative analysis of human societies (Moran 1995) and the science/research plan of the Land-Use and Land-Cover Change (LUCC) Project (Turner et al. 1995). Lack of progress toward this goal may be attributable to a number of factors, perhaps primordially the absence of a disciplinary home. By its nature, LCS is an interdisciplinary endeavor, with practitioners axiomatically operating at the edges of fields whose professional societies may not be motivated to develop or encourage the use of standard research protocols. Likewise, while journals devoted to land change topics have established footholds in the rapidly growing universe of peer-reviewed publications, none has yet developed procedures for describing, much less archiving, the data on which submissions are based. Some progress has been achieved through increasingly stringent requirements on the part of funding agencies (e.g., US NSF) that grant recipients make their data available, e.g., through the Inter-University Consortium for Political and Social Research (ICPSR); however, formal requirements have generally been limited to US examples. The development and widespread use of optimal meta-knowledge systems for LCS synthesis will require concerted efforts on the parts of professional societies, journals, and funding agencies and particularly the global change research community, including the Global Land Project.

### Case acquisition and selection bias

The first task of a meta-analyst is to define the phenomenon under study, a set of keywords that bound the population of case studies, the languages within which s/he wants to search, and a search and selection strategy. While English is often the default option, particular topics merit searches across a series of languages. A search of case studies of tropical deforestation, for example, will be more complete if it includes journals published in French, Portuguese, and Spanish. Key word searches in the Web of Science^®^, and other databases can establish the population of case studies for analysis. Additionally, a scoping search prior to a full search helps to select appropriate sources and refine research questions and keywords. Using search engines and literature databases certainly identifies a population of scientifically reputable studies, but it can miss studies in the gray literature, such as government reports. This is often referred to as the ‘file drawer problem’ in which the population of studies used in meta-analyses tend to be those that are readily accessible, which biases the selection of studies by neglecting ‘gray literature’ and non-English publications. Incorporation of this gray literature into meta-studies has become increasingly unlikely due to the ease of generating the population of cases for study through search engines that focus on peer-reviewed literature. Additional case studies outside of the coverage of search engines can be included through expert recommendation. Although this may increase the number of relevant case studies considered, it may also introduce other biases into the case selection process and reduce the repeatability of the meta-study.

Another source of bias is introduced because case-study topics and locations often follow ‘fashion trends,’ and researchers tend to study negative rather than positive developments. An example is the Sahelian region of sub-saharan Africa, where the vast majority of case studies described processes, drivers, and impacts of desertification as being mainly human-driven (e.g., as outlined in Mortimore and Turner 2005) despite a counter literature that challenged this notion (Nicholson et al. 1990) and others providing evidence that desertification was not as widespread as previously thought and a ‘greening’ of the region was even observed (e.g., Olsson et al. 2005; Rasmussen et al. 2001). The focus on desertification was rooted in the fact that it was an issue high on the policy agenda, and hence, funding was available to study it and case-study sites were selected in areas where desertification was likely to be found (Mortimore and Turner 2005; Rasmussen et al. 2001). This example is not unique and can only be addressed if local case-study selection is done such that the full range of variation possible in the land change phenomenon of interest is represented. Defining and explicitly presenting precise case selection criteria will make clear the intended scope of the meta-study and allow reselection of the case set used for analysis.

It can also be difficult to obtain—or even know—the full range of variation in land changes because locales without the land changes of interest do not attract investigator interest and, as a result, the dynamics that contribute to no change may not be well represented in the published literature. This problem can be overcome, to some degree, by searching for detailed ethnographic studies of peoples who inhabit the areas of interest. While these studies may mention land-use change almost as an aside, they can provide valuable contrasts, when coded, to those cases that report an abundance of change. Similarly, case studies which did not find significant effects of land change are less likely to be published and thus might cause a bias in meta-analyses of effect sizes (e.g., Gurevitch and Hedges 1999). However, it is possible to detect this publication bias and quantify its impact on the validity of the results (Gurevitch and Hedges 1999; Nakagawa and Santos 2012).

Prospects for enhancing the availability of case studies lie in the data from local case studies being recorded and stored in a more accessible manner. Many valuable case-study results remain in unpublished theses and gray literature for a variety of reasons: They are never submitted for peer-reviewed publication because language barriers or lack of incentives to publish internationally; they are difficult to publish because they replicate other studies and/or lack significant findings of change (i.e., ‘no change’ case studies); and their data may have been produced for specific development projects, and access is therefore restricted. Efforts to make such studies available would require considerable efforts in obtaining access to reports and theses in multiple locations and languages. Translating published case-study literature into English and sharing it online would also vastly expand the geographic coverage and amount of case-study research available. Infrastructure that addresses these potentially large and rich data sources is highly valuable (e.g., the Inter-university Consortium for Political and Social Research, http://www.icpsr.umich.edu).

## Conclusions

Synthesis studies in LCS have rapidly increased over the last decade and will undoubtedly remain essential for generating systematic understanding of local land change processes globally. Due to the complex, multicausal nature of land change, synthesis in LCS requires a diverse suite of synthesis and meta-study methods that can cope with multiscale causes and consequences, integration of quantitative and qualitative data, and uneven analytical and reporting standards among contributing disciplines. Key challenges of data standardization, interpretability, and selection bias across case studies remain. Addressing these shortcomings will require coordination between case-study and meta-study researchers within the LCS community. The availability and representativeness of case studies will remain a constraint for global and regional synthesis in LCS as long as new case studies are produced without consideration of broader needs for understanding land change at regional or global level. Synthesis methods, and meta-studies in particular, could address this limitation by reporting on knowledge gaps observed in the synthesis process, helping to guide the production of new case studies toward understanding specific problems and regions in a more systematic manner.

Similarly, standards for conducting and reporting synthesis research in LCS could be advanced by adopting practices like those developed in other disciplines but with some enhancements specific to LCS. Such standards could clearly signal data and spatial information requirements to case-study researchers, which may improve consistency and comparability across case studies and facilitate synthesis. Perhaps the simplest of these is the adoption of standards for meta-study analysis and publication, such as the PRISMA statement, which demands that essential criteria for conducting meta-studies be reported in all published work (e.g., number of studies found/rejected, criteria for rejection, etc., Liberati et al. 2009). A more ambitious example is provided by bioinformatics, which emerged as a powerful synthetic discipline by combining specialized cyber-infrastructures and data standards enabling rapid data sharing, searching, and synthesis (e.g., genbank; http://www.ncbi.nlm.nih.gov/nucleotide) coupled with a new culture of scientific data sharing (Kaye et al. 2009).

Advancing synthesis in LCS will require bolstering synthesis efforts by individual researchers and their mentoring of new students toward synthetic research, complemented by the efforts of scientific societies, funding agencies and publishers, in part by implementing research sharing conventions and in part by instilling the informal professional pressures that could help to establish and enforce ‘best practices’ for sharing. In LCS, the Global Land Project (www.globallandproject.org) is particularly well positioned to advance a culture of scientific data sharing, including recent efforts of the GLOBE project (Ellis 2012; Young et al. 2013; http://globe.umbc.edu) to create an open online database of user-contributed and geographically described case studies coupled with tools for searching, grouping, and assessing the global relevance of studies based on their geographic context using a geo-social–computational system. GLOBE and other geo-cyberinfrastructure efforts have the potential to move LCS as a discipline toward more effective global and regional observational strategies based on quantifying global knowledge gaps in local studies in order to inform the selection of sites for future research. Ultimately, however, the prospect of advancing synthesis in LCS will depend on the coordinated efforts of the LCS community to improve the effectiveness of data sharing and the process of knowledge generation by both case-study and meta-study researchers.

## Electronic supplementary material

Below is the link to the electronic supplementary material.

Appendix 1 (DOCX 15 kb)

Appendix 2 (XLSX 4853 kb)
