# Supplementary material for: Synthesis in land change science: methodological patterns, challenges, and guidelines
Source: Reg Environ Change. 2014 Jun 6;15(2):211–26. doi: 10.1007/s10113-014-0626-8 (PMC4372122; doi:10.1007/s10113-014-0626-8)
Supplement: Supplementary file 1 — Appendix 1 (DOCX 15 kb) [file 10113_2014_626_MOESM1_ESM.docx]

**Appendix A**

*A1.1. Target set of key LCS meta-studies:*

Angelsen, A. and Kaimowitz, D. (1999) Rethinking the causes of deforestation: Lessons from Economic Models. World Bank Res. Obser. 14(1), 73-98.

Brown, D.G., Johnson, K.M., Loveland, T.R., and Theobald, D.M. (2005) Rural land-use trends in the conterminous United States, 1950-2000. Ecol. Appl. 15(6), 1851-1863.

Geist, H. and Lambin, E. (2004) Dynamic causal patterns of desertification. Bioscience 54(9), 817.

Gibbs, HK. et al. (2008) Carbon payback times for crop-based biofuel expansion in the tropics: the effects of changing yield and technology. Environ. Res. Lett. 3, 1-10.

Guo, LB & Gifford, RM (2002) Soil carbon stocks and land use change: a meta analysis. Glob. Change Biol. 8(4), 345-360.

Keys, E. and McConnell, W.J. (2005) Global change and the intensification of agriculture in the tropics. Glob. Environ. Change 15, 320-337.

Kull, C.A. et al. (2011) Adoption, use and perception of Australian acacias around the world. Divers. Distrib. 17, 822-836.

Ladha, J.K., Reddy, C.K., Padre, A.T., and van Kessel, C. (2011) Role of nitrogen fertilization in sustaining organic matter in cultivated soils. J. of Environ. Qual. 40(6), 1756-1766.

Misselhorn, A. (2005) What drives food security in southern Africa?: a meta-analysis of household economy studies. Glob. Environ. Change 15, 33–43.

Oldekop, J.A., Bebbington, A.J., Brockington, D., and Preziosi, R.F. (2010) Understanding the lessons and limitations of conservation and development. Conserv. Biol. 24(2), 461-469.

Pagdee, A., Kim, Y., Daugherty, P. (2006) What makes community forest management successful: a meta-study from community forests throughout the world. Soc. Natur. Resour. 19, 33–52.

Rudel, T.K. (2007) Changing agents of deforestation: From state-initiated to enterprise driven processes, 1970-2000. Land Use Policy 24(1), 35-41.

Scanlon, B.R. et al. (2006) Global synthesis of groundwater recharge in semiarid ad arid regions. Hydrol. Process. 20(15), 3335-3370.

Schueler, T.R., Fraley-McNeal, L., Cappiella, K. (2009) Is impervious cover still important? Review of recent research. J. Hydrol. Eng. 14(4), 309.

Seto, K.C., Fragkias, M., Güneralp, B., Reilly, M.K. (2011) A Meta-Analysis of Global Urban Land Expansion. PLoS ONE 6(8): e23777. doi:10.1371/journal.pone.0023777

Syers, J.K, Lingard, J., Pieri, C., Ezcurra, E., and Faure, G. (1996) Sustianable land management for the semiarid and sub-humid tropics. Ambio 25(8), 484.

Thiam, A., Bravo-Ureta, B.E., Rivas, T.E. (2005) Technical efficiency in developing country agriculture: a meta-analysis. Agric. Econ. 25(2-3), 235-243.

Tonitto, C., David, M.B., Drinkwater, L.E. (2005) Replacing bare fallows with cover crops in fertilizer-intensive cropping systems: A meta-analysis of crop yield and N dynamics. Agric. Ecosys. Environ. 112(1), 58-72.

van Vliet, N., Mertz, O., Heinimann, A. (2012) Trends, drivers and impacts of changes in swidden cultivation in tropical forest-agriculture frontiers: A global assessment, Glob. Environ. Change 22(2), 418-429.

*A1.2 Search keywords and filters*

TS=(((meta AND (study OR analy*)) OR "case studies" OR synthes* OR intergrat* OR (meta- AND (study OR anal*)) OR (geographic* AND database)) AND ((land AND (use OR cover)) OR (land- AND (use OR cover)) OR "food *security" OR ((environmental OR forest OR land OR resource* OR "common pool" OR (agricultur* AND production)) AND (management OR economics OR cultivat*))) AND (((proximate OR direct OR indirect) AND causes) OR "driv*" OR crop* OR urban OR rural OR deforestation OR agricultur* OR forest OR dryland OR desertification OR expansion OR intensification OR conservation))

Refined by: Web of Science Categories=(ECOLOGY OR ENVIRONMENTAL SCIENCES OR ENVIRONMENTAL STUDIES OR WATER RESOURCES OR PLANT SCIENCES OR BIODIVERSITY CONSERVATION OR AGRICULTURE MULTIDISCIPLINARY OR GEOGRAPHY OR SOIL SCIENCE OR METEOROLOGY ATMOSPHERIC SCIENCES OR PLANNING DEVELOPMENT OR AGRICULTURAL ECONOMICS POLICY OR ECONOMICS OR ENGINEERING CIVIL OR SOCIOLOGY OR MULTIDISCIPLINARY SCIENCES OR BIOLOGY )
